# Metabolic syndrome and its components among HIV/AIDS patients on Antiretroviral Therapy and ART-Naïve Patients at the University of Calabar Teaching Hospital, Calabar, Nigeria

**DOI:** 10.4314/ahs.v22i1.50

**Published:** 2022-03

**Authors:** Ebot Ojong, Bassey Iya, Jules Djeufouata, Forwah Ndeh, Augusta nsonwu, Vigny Njongang, Maisie Etukudo, Chinyere Usoro, Julie Ekpo

**Affiliations:** 1 University of Buea, Medical Laboratory Sciences; 2 University of Calabar, Nigeria, Medical Laboratory Sciences, Faculty of Allied Medical Sciences; 3 University of Buea, Cameroon, Medical Laboratory Sciences; 4 University of calabar, department of medical laboratory science; 5 University of Buea, Cameroon, Medical Laboratory Sciences; 6 University of Calabar, Nigeria, Medical Laboratory Sciences, Faculty of Allied Medical Sciences; 7 University of Calabar, Nigeria, Medical Laboratory Sciences; 8 Akwa Ibom State Hospital Management Board, Uyo, Nigeria, Akwa Ibom State Hospital Management Board

**Keywords:** HIV/AIDS patients, antiretroviral therapy, ART-Naïve patients, Calabar Teaching Hospital, Calabar, Nigeria

## Abstract

**Background:**

Although an increasing access to ART in sub-Saharan Africa has made it possible for HIV/AIDS patients to live longer, clinicians managing such patients are faced with the challenge of drug-related metabolic complications.

**Methods:**

A cross -sectional study was carried out at the University of Calabar Teaching Hospital, Nigeria, on three groups of participants; namely HIV patients on ART, ART-naïve patients and HIV negative subjects (n =75). Demographic and anthropometric data were collected using a well-structured questionnaire while biochemical parameters were measured using colorimetric methods.

**Results:**

The highest prevalence of MS was associated with the HIV/AIDS patients on ART (i.e. 32.0 %, and 50.3% for NCEP-ATP III and IDF criteria respectively). Patients on ART had significant increases (p< 0.05) in waist to hip ratio, FPG, serum TG and LDL-c; and a significantly higher (p< 0.05) prevalence of hypertension, diabetes, low HDL-c and hypertriglyceridaemia compared to the ART-naïve patients. Low serum HDL-c was the most prevalent form of dyslipidaemia in all three groups and the most prevalent component of MS in HIV patients.

**Conclusion:**

ART increases the risk of MS and CVD. HIV/AIDS patients on ART should be advised on lifestyle modifications and undertake regular assessment of their cardiovascular risk factors.

## Introduction

For almost four decades, the impact of HIV/AIDS on demographic, social and economic conditions has been substantial^1^. In 2019, UNAIDS estimated that 64% of all daily new HIV infections occurred in sub-Saharan Africa. The first two cases of HIV/AIDS in Nigeria were identified in 1985^2^. In 2019, Nigeria had a national HIV prevalence of 2.8%. 100 000 people were newly infected with HIV and 45 000 people died from an AIDS-related illness. About 65% of people living with HIV in Nigeria were accessing antiretroviral therapy in 2019^1^. Although the medical management of HIV has been revolutionized by the use of ART, drug-related metabolic complications continue to constitute a major challenge to clinicians in the management of HIV/AIDS^3^. HIV infection and ART can induce lipodystrophy, insulin resistance and dyslipidaemia which are risk factors of metabolic syndrome^3^.

Metabolic syndrome has received much attention in recent times due to increasing awareness of its association with a 5-fold risk of type 2 diabetes mellitus and a 2-fold risk of cardiovascular disease^4^. Global prevalence of metabolic syndrome range from 17.0% to 45.4% while reported prevalence in Nigeria range from 11.0% to 21.0% in HIV/AIDS patients on ART and 2.0% to 9.0% in ART- naïve individuals^5^,^6^. Dietary and lifestyle choices and a combination of genetic, cultural and environmental factors may contribute to the differences in this observed prevalence^7^. Several definitions have been put forward by different expert groups, defining metabolic syndrome from different perspectives with no unified definition adopted. In the present study, the National Cholesterol Education Program-Adult Treatment Panel III 2001 criteria (NCEP ATP-III)^8^ and the International Diabetes Federation (IDF)^9^ criteria were used to determine the prevalence of MS and its components among HIV/AIDS patients. The IDF criteria, the most recent definition of the MS have identified the need for research in epidemiological, clinical and biochemical characterization of MS9. The objective of this study was to determine and compare the prevalence of metabolic syndrome and its components among HIV/AIDS patients and ART-naïve patients with healthy control subjects.

## Methods

### Study design and study area

This was a cross-sectional study conducted at the President's Emergency Plan for AIDS Relief (PEPFAR) Clinic of the University of Calabar Teaching Hospital, Calabar, Nigeria from January 2017 to February, 2018. Calabar lies in latitude 4°57′0″N and longitude 8°19′30″E and is located 604 km from Abuja, Nigeria's capital^10^.

### Study population and data collection

Participants included seventy five newly diagnosed ART-naïve (untreated) HIV seropositive individuals, seventy five HIV positive patients on uninterrupted ART for at least six months, and seventy five apparently healthy HIV negative controls, selected by stratified random sampling. The sample size of 150 HIV/AIDS patients was calculated from reported prevalence of metabolic syndrome in HIV/AIDS patients5,6. Patients were enrolled into the study if they had HIV infection and were on ART for at least six months (ART group) or were not on ART (ART-naïve group). Patients were not included in the study if diabetes mellitus, hypertension, dyslipidaemia, coronary artery disease, malignancy, sleep apnoea, or chronic renal failure were present prior to starting ART or if they were non-adherent to ART. Face to face interviews were conducted using a well-structured questionnaire. Diastolic and systolic blood pressure, body weight and height were measured using standard procedures. Body mass index was calculated for each participant as weight in kilogram (kg) divided by the height in meter squared (m^2^) and expressed in kilograms per square metre (kg/m^2^). The waist to hip ratio was calculated by dividing the waist circumference by the hip circumference.

### Sample collection and laboratory analysis

Five mls of venous blood was aseptically collected from study participants after an overnight fast of at least 12 hours. Two mls of blood was dispensed into fluoride oxalate tubes for fasting plasma glucose determination and the rest into plain tubes for measurement of other biochemical parameters. A rapid test to screen for the presence of HIV antibodies was performed using the rapid test kit, DetermineTM (Abbot Laboratories, Tokyo, Japan). All positive tests were confirmed with the ImmunoComb II HIV 1 and 2 Bispot kit (PDS - Orgenics, Israel). Total CD4+ T cell counts were performed using the Partec CyFlow Counter, using the flow cytometry technique. Fasting plasma glucose concentration was determined by the glucose oxidase method. Total serum cholesterol concentration was determined by an enzymatic colorimetric method using commercially available kits from ELITech Clinical Systems, France. Triglycerides concentration was determined by an enzymatic colorimetric method. High density lipoprotein cholesterol was measured using commercially available kits from Giesse Diagnostics, Rome, Italy. The low density lipoprotein cholesterol (LDL-c) concentration in serum was calculated according to Friedewald's equation11. Control samples were supplied with the ELISA kits while commercially available controls were included in the other tests.

### Diagnostic criteria for the metabolic syndrome

The NCEP-ATP III Criteria diagnose MS if three or more of the following five components of metabolic syndrome were present: abdominal obesity (waist circumference >102cm or 40 inches for men or >88cm or 36 inches for women); dyslipidaemia – (raised triglyceride ≥ 150mg/dl (1.7mmol L-1), reduced HDL-c < 40mg/dl (0.9mmol L-1) in men or < 50mg/dl (1.0mmol L-1) in women); high blood pressure (systolic BP ≥130mmHg or diastolic BP ≥85mmHg or treatment of hypertension) and raised fasting glucose (≥6.1mmol L-1).

According to the IDF, MS was diagnosed if central obesity (Body Mass Index (BMI) >30kg/m2, waist circumference >90cm for men or >80cm for women) was accompanied by any two of the following four factors: raised triglycerides level ≥150mg/dl (1.7 mmol L-1); reduced HDL-c <40mg/dl (1.03 mmol L-1) for men or <50mg/dl (1.29mmol L-1) for women; raised blood pressure: systolic BP>130 mmHg or diastolic BP>85mmHg or treatment of previously diagnosed hypertension and a raised fasting blood glucose (FBS) ≥100mg/dl (5.6mmol L-1) or previously diagnosed type 2 diabetes.

### Ethical considerations

This study was approved (Ref: CRS/MH/HREC/015/Vol.V/172) by the Cross River State - Health Research Ethics Committee (CRS-HREC) Calabar, Nigeria. All information obtained from participants was kept with utmost confidentiality.

### Statistical analysis

Data were analyzed using the statistical package for social sciences for windows (SPSS), software version 18.0. Results were reported as mean±SD. Differences between group means were compared using the student's T-test or analysis of variance (ANOVA) and Pearson's Chi- Square test, since the data followed a normal distribution. Tukey's Post hoc tests were run to confirm where the differences occurred between groups. Statistical significance was set at P ≤ 0.05.

## Results

The study participants were distributed into three cohorts where 33.33% (75) were HIV positive patients on ART, 33.33% (75) were ART – naïve HIV patients and 33.33% (75) constituted the HIV negative control group. Among the participants, 48.9% (110) were male and 51.1% (115) were female. The mean age of participants was 39.20 years, median was 37.00 years.

The prevalence of components of metabolic syndrome using the NCEP ATP -III criteria is shown in Table 1. The prevalence of hypertension, diabetes, low HDL-c and hypertriglyceridaemia were significantly higher (p< 0.05) in both HIV groups compared to the controls. Patients on ART showed significantly higher (p< 0.05) prevalence of low HDL-c and hypertriglyceridaemia compared to the ART-naïve patients (Table 1). Based on the IDF criteria, patients on ART exhibited significantly higher (p< 0.05) prevalence of hypertension, diabetes, low serum HDL-c and hypertriglyceridaemia compared to the ART-naïve patients (Table 2). Low serum HDL-c was observed to be the most prevalent component in HIV patients on ART while central obesity was the most prevalent component in ART – naïve patients and controls (Table 2).

**Table 1:** Components of metabolic syndrome in HIV positive patients on ART, ART- naïve patients and controls based on NCEP ATP -III criteria

Risk factors for metabolic syndrome based on NCEP ATP – III criteria	HIV positive on ART n = 75 n (%)	HIV positive ART-Naive n = 75 n (%)	Controls n = 75 n (%)	χ^2^	p-value
Hypertension (130/85mmHg)	22(29.3)	18(24.0)	0(0.0)	25.063	0.000*
Diabetes (FPG ≥ 6.1 mmol/l)	19(25.3)	16(21.3)	0(0.0)	10.125	0.000*
Low HDL cholesterol(< 0.9 mmol/l in men or < 1.0 mmol/l inwomen)	64(85.3)	33(44.0)	17(22.7)	58.753	0.000*
Hypertriglyceridaemia (≥ 1.70 mmol/l)	29 (38.6)	14(18.6)	7(9.3)	17.880	0.000*
Waist circumference (> 102cm in men or >88cm in women)	23(30.6)	25(33.3)	18(24)	2.691	0.261

* Statistically significant, FPG = fasting plasma glucose, HDL = high density lipoprotein, ART = antiretroviral therapy, n = number of subjects studied

**Table 2:** Components of metabolic syndrome in HIV positive patients on ART, ART-naïve patients and controls based on the IDF criteria

Risk factors for metabolic syndrome based on IDF criteria	HIV positive on ART n = 75 n (%)	HIV positive ART-Naive n = 75 n (%)	Controls n = 75 n (%)	χ2	p-value
Hypertension (130/85mmHg)	22(29.3)	18(24.0)	0(0.0)	59.187	0.000*
Diabetes (FPG ≥ 5.6 mmol/l)	34(45.3)	20(26.6)	0(0.0)	41.042	0.000*
Low HDL cholesterol(< 1.03 mmol/l in men or 1.29 mmol/l in women)	68 (90.6)	39(52.0)	32(42.7)	45.506	0.000*
Hypertriglyceridaemia (≥ 1.70 mmol/l)	29(38.6)	14(18.6)	7(9.33)	17.880	0.000*
Waist circumference (> 90cm in men and 80cmin women)	50(66.6)	43(57.3)	39(52.0)	7.373	0.026*

* Statistically significant, FPG = fasting plasma glucose, HDL = high density lipoprotein, ART = antiretroviral therapy, n = number of subjects studied

As shown in Table 3, the prevalence of metabolic syndrome in HIV positive patients on ART, ART-naïve HIV patients and controls using the NCEP -ATP III criteria were 32.0%, 6.7% and 2.7% respectively. Using the IDF criteria, the prevalence were 50.3%, 20.0% and 8.0% respectively. The prevalence was significantly higher (p< 0.05) in both HIV groups compared to the controls when the NCEP -ATP III and IDF criteria were used. HIV positive patients on ART showed a higher (p< 0.05) prevalence of metabolic syndrome compared to the HIV positive ART-naïve patients using both classifications (Table 3). The prevalence of MS in all three groups based on IDF was significantly higher (P<0.05) compared to that obtained using NCEP -ATP III standard.

**Table 3:** Prevalence of metabolic syndrome in tests and control subjects based on NCEP ATP -III and IDF criteria

Group	NCEP -ATP III n(%)	IDF n (%)	χ2-test	p-value
On ART n = 75	24(32.0)	40(50.3)	11.279	0.001*
ART - naive n = 75	5(6.7)	18(20.0)	16.964	0.000*
Control n = 75	2(2.7)	6(8.0)	16.281	0.000*
Total	18(8.0)	64(28.4)	42.897	0.000*

* Statistically significant

ART = antiretroviral therapy, n = number of subjects studied

The control subjects had the highest mean value of high density lipoprotein cholesterol and CD4+ cell count among the three groups (Table 5). The HIV patients on ART had the highest mean values of fasting plasma glucose, triglycerides, total cholesterol, low density lipoprotein cholesterol and waist: hip ratio among the three groups (Table 4, 5). They also had lower (p< 0.05) mean values of high density lipoprotein cholesterol compared to the ART – naïve patients (Table 6). The ART – naïve group had significantly higher (p< 0.05) mean values of fasting plasma glucose (p< 0.05), waist: hip ratio than control subjects (Table 6).

**Table 5:** Biochemical parameters and CD4 cell count of study participants

Parameters	HIV patients on ART n = 75	ART – naive HIV patients n = 75	Controls n = 75	Calc. F	Crit. F	p- value
FPG (mmol/L)	5.80±1.28	5.55±1.99	4.70±0.71	8.944	3.020	0.000*
TG (mmol/L)	1.77±1.34	1.37±0.83	1.04±0.56	8.387	3.020	0.000*
TC (mmol/L)	3.93±1.15	3.79±1.17	3.81±1.20	0.298	3.020	0.743
HDL-c (mmol/L)	0.63±0.32	1.24±0.92	1.47±0.76	25.210	3.020	0.000*
LDL-c (mmol/L)	2.56±1.09	1.95±1.09	1.88±0.98	8.465	3.020	0.000*
CD4 count (cells/µL)	434.79±243.03	409.47±262.37	931.02±168.62	92.899	3.020	0.000*

FPG = fasting plasma glucose, TG = triglycerides, TC = total cholesterol, HDL-c = high density lipoprotein cholesterol, LDL-c = low density lipoprotein cholesterol, Calc. F = Calculated F, Crit. F = Critical F n = number of subjects studied, values are mean±SD

* statistically significant

**Table 4:** Demographic and anthropometric parameters of participants

Parameter	HIV patients on ART n = 75	ART – naïve HIV patients n = 75	Controls n = 75	Calc. F	Crit. F	p- value
Age (years)	39.96±9.12	37.84±9.54	38.42±8.67	0.397	3.020	0.534
Weight (kg)	66.08±11.73	64.48±12.99	66.23±10.42	0.463	3.020	0.630
Height (m)	1.64±0.08	1.63±0.09	1.63±0.09	0.953	3.020	0.661
BMI (kg/m^2^)	24.77±4.05	25.22±5.11	25.34±4.78	0.272	3.020	0.762
WC (cm)	89.25±9.52	85.15±18.19	83.51±18.09	2.417	3.020	0.092
HC (cm)	102.08±8.79	101.89±15.99	104.47±19.79	0.540	3.020	0.583
W/H	0.87±0.10	0.84±0.08	0.80±0.07	8.748	3.020	0.000*
SBP (mmHg)	128.17±17.98	125.36±16.60	125.23±13.56	0.723	3.020	0.486
DBP (mmHg)	84.55±14.79	82.08±11.20	83.89±10.86	0.762	3.020	0.468

BMI = body mass index, WC = waist circumference, HC = hip circumference, W/H = waist: hip ratio, SBP = systolic blood pressure, DBP = diastolic blood pressure, ART = antiretroviral therapy, Calc. F = Calculated F, Crit. F = Critical F, n = number of subjects studied, values are mean±SD

* statistically significant.

**Table 6:** Comparison of anthropometric and biochemical parameters among study participants in post hoc analysis

Parameter	Mean Difference	p-value
HIV patients on ART and ART .naive HIV		
HDL-c (mmol/L)	0.609	0.0001*
LDL-c (mmol/L)	0.612	0.002*
TG (mmol/L)	0.402	0.039*

ART – naive HIV patients and controls		
Waist/Hip Ratio	0.045	0.017*
FPG (mmol/L)	0.853	0.005*
CD4 (cells/µL)	521.552	0.0001*

controls and HIV patients on ART		
Waist/Hip Ratio	0.068	0.0001*
FPG (mmol/L)	1.099	0.0001*
HDL-c (mmol/L)	0.841	0.0001*
LDL-c (mmol/L)	0.680	0.0001*
TG (mmol/L)	0.724	0.0001*
CD4 (cells/µL)	496.232	0.0001*

* statistically significant

## Discussion

It has been reported that, there is no clear pattern in the frequency of the different components of MS from previous studies in African adults 12. The finding of central obesity and hypertension as two of the most prevalent components of MS in the ART group in the present study is consistent with other reports 6, 13. It has been postulated that untreated HIV disease may tend to lower blood pressure, while normalisation of immune status and suppression of HIV replication with potent antiretroviral combinations elevate blood pressure 14

The prevalence of MS varies according to the criteria used and other variables such as gender, age, race, socioeconomic status, work – related activities, ethnicity, location and disease condition of the population studied^15^. The prevalence of MS of 50.3% in HIV patients on ART using the IDF guideline observed in the present study is higher than that reported by a 12-year systematic review for the general population in Nigeria which is 38.4% 16. Higher prevalence of MS in patients on ART compared to ART- naïve patients have also been reported in studies in Cameroon and Northern Nigeria 6, 17.

However, reports from a study in South Eastern Nigeria suggested that HIV-infection in South East Nigeria is not associated with a higher prevalence of MS^18^. The finding of a higher prevalence of MS using the IDF criteria corroborates other studies conducted in Europe, Nigeria and Cameroon 10, 17, 19

In contrast, two studies in Nigeria documented that there was no significant difference between the prevalence of MS using NCEP ATP-III and IDF definitions among native Abuja settlers and hypertensive patients^20^. Although several studies have found an increase in CD4 counts with ART use there was no significant difference in the mean CD4 counts of ART -naïve and ART treated patients in the present study 21. This disparity may be explained by the fact that the ART- naïve participants were recruited during a voluntary HIV testing campaign in Calabar and did not present with opportunistic infections like tuberculosis and HIV wasting syndrome common in HIV/AIDS patients 22.

Long-term use of ART has been associated with a number of metabolic complications such as dysglycaemia, insulin resistance, dyslipidaemia and lipodystrophy 23.

The finding of higher prevalences of dyslipidaemia, dysglycaemia and hypertension in the ART group in the present study is in line with similar observations made by Dimodi et al, 17. Changes in lipid profile parameters in HIV infection and ART use have been reported by several studies^24^.

## Conclusion

The prevalence of MS based on the IDF criteria is higher when compared to that obtained using the NCEP ATP-III standard. The metabolic syndrome is associated with both HIV infection and use of ART. However, ART treated HIV patients have a significantly higher burden of metabolic syndrome and cardiovascular disease risk factors compared to treatment-naïve patients. HIV/AIDS patients on ART should be advised on lifestyle adjustments to prevent the development of metabolic syndrome.
